# Efficacy and Limitations of Chemically Diverse Small-Molecule Enzyme-Inhibitors against the Synergistic Coagulotoxic Activities of *Bitis* Viper Venoms

**DOI:** 10.3390/molecules27051733

**Published:** 2022-03-07

**Authors:** Nicholas J. Youngman, Matthew R. Lewin, Rebecca Carter, Arno Naude, Bryan G. Fry

**Affiliations:** 1 Venom Evolution Lab, School of Biological Science, University of Queensland, St. Lucia, QLD 4072, Australia; 2 California Academy of Sciences, San Francisco, CA 94118, USA; matt@ophirex.com; 3 Ophirex Inc., Corte Madera, CA 94925, USA; rebecca@ophirex.com; 4 Snakebite Assist, Pretoria ZA-0001, South Africa; afnaude@worldonline.co.za

**Keywords:** venom, anticoagulant, *Bitis*, PLA_2_, varespladib, prinomastat, marimastat

## Abstract

Snakebite remains a significant public health burden globally, disproportionately affecting low-income and impoverished regions of the world. Recently, researchers have begun to focus on the use of small-molecule inhibitors as potential candidates for the neutralisation of key snake venom toxins and as potential field therapies. *Bitis* vipers represent some of the most medically important as well as frequently encountered snake species in Africa, with a number of species possessing anticoagulant phospholipase A_2_ (PLA_2_) toxins that prevent the prothrombinase complex from inducing clot formation. Additionally, species within the genus are known to exert pseudo-procoagulant activity, whereby kallikrein enzymatic toxins cleave fibrinogen to form a weak fibrin clot that rapidly degrades, thereby depleting fibrinogen levels and contributing to the net anticoagulant state. Utilising well-validated coagulation assays measuring time until clot formation, this study addresses the in vitro efficacy of three small molecule enzyme inhibitors (marimastat, prinomastat and varespladib) in neutralising these aforementioned activities. The PLA_2_ inhibitor varespladib showed the greatest efficacy for the neutralisation of PLA_2_-driven anticoagulant venom activity, with the metalloproteinase inhibitors prinomastat and marimastat both showing low and highly variable degrees of cross-neutralisation with PLA_2_ anticoagulant toxicity. However, none of the inhibitors showed efficacy in neutralising the pseudo-procoagulant venom activity exerted by the venom of *B. caudalis*. Our results highlight the complex nature of snake venoms, for which single-compound treatments will not be universally effective, but combinations might prove highly effective. Despite the limitations of these inhibitors with regards to in vitro kallikrein enzyme pseudo-procoagulant venom activity, our results further support the growing body of literature indicating the potential use of small molecule inhibitors to enhance first-aid treatment of snakebite envenoming, particularly in cases where hospital and thus antivenom treatment is either unavailable or far away.

## 1. Introduction

Snakebite envenoming remains a neglected health issue, particularly in tropical and subtropical countries around the world, with approximately 5.4 million snakebites and up to 140,000 deaths occurring annually [1,2,3]. However, these numbers are well-recognised as gross underestimations due to the poor to non-existent record keeping in the most affected regions. The continent of Africa is a region particularly affected by snakebites, with envenomings by *Bitis* species representing one of the most significant causes of snakebite mortality and morbidity throughout the region [2,3,4]. *Bitis arietans* is the most medically significant species within the genus and thus has received the most attention [5,6,7,8,9]. However, a number of smaller species significantly contribute to the morbidity of snakebites across Africa, with the species *B. atropos* (Eswatini, Lesotho, Mozambique, South Africa and Zimbabwe), *B. caudalis* (Angola, Botswana, Namibia, South Africa and Zimbabwe) and *B. cornuta* (Namibia and South Africa) all capable of inflicting severe envenomings [9,10,11,12].

The species *B. atropos*, *B. caudalis* and *B. cornuta* are all a part of the evolutionary clade of dwarf *Bitis* within the subgenera *Calechidna*, which are restricted to southern Africa [13,14]. Due to the complex toxin diversity within *Bitis* venoms and, in particular, dwarf *Bitis*, envenomings can result in a wide range of clinical pathologies, including: severe swelling, tissue necrosis, hypotension, severe coagulopathy, hyponatremia, dysphagia and respiratory failure [10,11]. In addition, *B. atropos*, *B. caudalis* and *B. cornuta* all possess snake venom phospholipase A_2_ (sPLA_2_) toxins, which exert anticoagulant activity through the inhibition of the prothrombinase complex, impeding its ability to induce clot formation by converting prothrombin to thrombin [15]. This activity is also highly variable across the allopatric populations of *B. atropos* and *B. caudalis*, particularly across *B. caudalis,* as some populations possess venom with a greater proportion of snake venom kallikrein-type serine proteinases, which deplete fibrinogen levels by directly cleaving fibrinogen in a pseudo-procoagulant manner to induce the formation of weak fibrin clots with short half-lives [15].

Although previous research has indicated that the South African Institute for Medical Research (SAIMR) polyvalent antivenom is effective in vitro at neutralising the anticoagulant and pseudo-procoagulant activities of these venoms [15], envenomings often occur in remote regions where the nearest hospital possessing antivenom is likely either great distances away or entirely unavailable. Due to the limitations of antivenom treatment, such as cold-storage requirements and the need for administration by trained health professionals due to the potential for adverse side effects such as allergic reaction, there remains a substantial need for easily administrable field-based treatments [16]. Small-molecule enzyme inhibitors have begun to be heavily investigated with respect to their suitability as broad-scale inhibitors for first-aid management following snakebites [17,18,19]. The PLA_2_ inhibitor varespladib has previously been shown to be a strong candidate in successfully neutralising sPLA_2_ toxins from a wide range of venomous snakes [17,20,21,22,23], including the anticoagulant activities of *Bitis* species [24]. Recent research has also indicated that some metalloproteases inhibitors show cross-neutralisation of sPLA_2_ -driven venom activities at concentrations similar to those in which they neutralise metalloprotease enzymatic toxins [25,26], which further highlights their potential use as field-based treatment options.

The aim of this study was to further investigate the efficacy of varespladib at neutralising the anticoagulant activity of *B. atropos*, *B. caudalis* and *B. cornuta* and to assess the relative cross-neutralising efficacy of two biochemically different metalloprotease inhibitors (marimastat and prinomastat) in comparison to varespladib. The efficacy of these three inhibitors at neutralising pseudo-procoagulant action, which is also present in the venom of *B. caudalis,* was also assessed. This study further explores and builds upon the growing body of literature concerning the suitability of small-molecule inhibitors (SMIs) for the treatment of snakebites.

## 2. Results and Discussion

**Figure 1:**
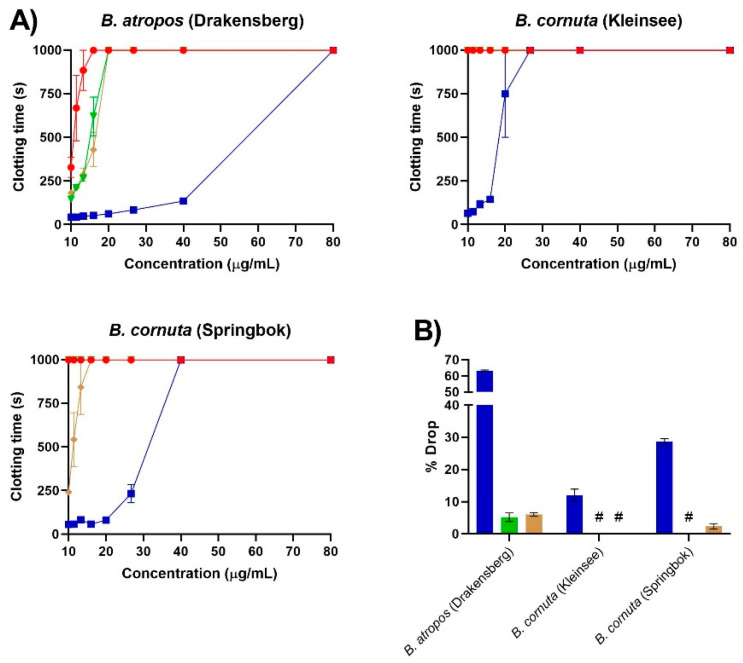
(**A**) Efficacy of varespladib, marimastat and prinomastat at neutralising the prothrombinase-inhibiting anticoagulant activity of *B. atropos* and *B. cornuta* venoms. Eight-point concentration curves, representing venom-induced clotting times (red circles), venom pre-incubated with varespladib-Na (blue squares), venom pre-incubated with marimastat (green triangles) and venom pre-incubated with prinomastat (brown diamonds). All values are mean ± SEM of *n* = 3. Note: some error bars are too small to see. (**B**) Percentage drop of plasma clotting time due to pre-incubation with either varespladib (blue), marimastat (green) or prinomastat (brown). All values are mean ± SEM of *n* = 3. # represents a 0% drop in venom activity. For all assays, there was a final reaction concentration of 0.4 nM (varespladib) and 0.2 mM (prinomastat and marimastat).

**Figure 2:**
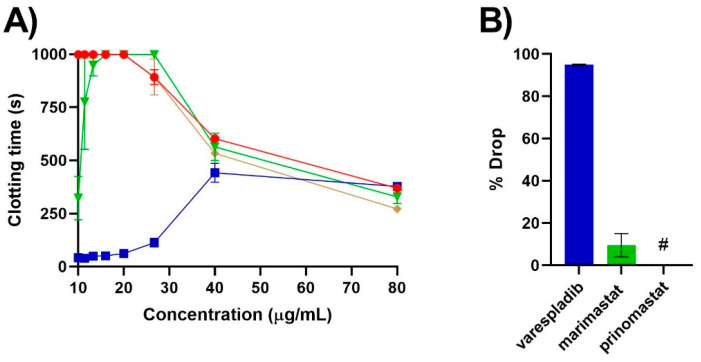
(**A**) Efficacy of varespladib, marimastat and prinomastat at neutralising the prothrombinase-inhibiting anticoagulant activity of *B. caudalis* (Rosh Pinah locality) venom. Eight-point concentration curve, representing venom-induced clotting times (red circles), venom pre-incubated with varespladib-Na (blue squares), venom pre-incubated with marimastat (green triangles) and venom pre-incubated with prinomastat (brown diamonds). All values are mean ± SEM of *n* = 3. Note: some error bars are too small to see. (**B**) Percentage drop (calculated using the venom concentrations of 20 µg/mL and lower only to exclude the coagulant effect observed at venom concentrations greater than 20 µg/mL) of plasma clotting time due to pre-incubation with either varespladib (blue), marimastat (green) or prinomastat (brown). All values are mean ± SEM of *n* = 3. # represents a 0% drop in venom activity. For all assays, there was a final reaction concentration of 0.4 nM (varespladib) and 0.2 mM (prinomastat and marimastat).

Our results expand upon recent work regarding the cross-neutralising efficacy of metalloprotease inhibitors in neutralising the anticoagulant activity of sPLA_2_ toxins [25]. The in vitro prothrombinase-inhibiting anticoagulant activity of *B. atropos*, *B. caudalis* and *B. cornuta* venoms in this study was neutralised by varespladib (Figure 1 and Figure 2), which is consistent with previous findings [24]. In contrast, metalloprotease inhibitors were extremely variable in their cross-neutralisation of PLA_2_-driven anticoagulant toxicities. Only the prothrombinase-inhibiting anticoagulant activity of *B. atropos* was neutralised by both prinomastat and marimastat metalloprotease inhibitors (Figure 1). In comparison, venom from the Springbok locality of *B. cornuta* showed some neutralisation by only prinomastat (Figure 1), whereas Rosh Pinah locale *B. caudalis* venom by only marimastat (Figure 2). In contrast, the venom from the Kleinsee locality of *B. cornuta* was not neutralised by either prinomastat or marimastat (Figure 1B).

The differential cross-neutralising activity of marimastat and prinomastat with respect to their efficacy on *Bitis* venom was also highly variable compared to a previous study on venoms from the *Naja* species. Previous research by Chowdhury et al. (2021) showed a 40–60% reduction in the sPLA_2_-driven anticoagulant activity of venom from African spitting cobras of the genus *Naja* in the presence of varespladib and prinomastat at the same concentrations as used in our study [25]. In the same study, marimastat had a much greater range of effect than varespladib or prinomastat against *Naja* anticoagulant activity, ranging from a less than 10% drop in activity (*N. pallida*) to one greater than 40% (*N. mossambica*, *N. nigricincta* and *N. nubiae*) [25]. Conversely, in this study, the prothrombinase-inhibiting anticoagulant activity of the dwarf *Bitis* venoms was not reduced to the same degree by prinomastat or marimastat cross-neutralisation as seen in previous studies against *Naja* venoms. The anticoagulant activity of *B. atropos* showed a less than 10% drop in activity in the presence of prinomastat or marimastat, whereas varespladib caused over a 60% drop in activity (Figure 1B). Only the Springbok locality of *B. cornuta* showed a drop in activity in the presence of prinomastat, which was a less than 5% reduction and therefore likely not functionally significant (Figure 1B). For the prothrombinase-inhibiting anticoagulant activity of *B. caudalis,* however, prinomastat had no cross-neutralisation, whereas marimastat showed a low level (less than 10% drop in activity) of cross-neutralisation (Figure 2B).

Our results suggest that although these metalloproteases may exhibit cross-neutralisation of sPLA_2_ toxins, as shown by previous studies [25,26], this cross-neutralisation may be limited and not broadly applicable to all sPLA_2_ toxins. As these *Bitis* venoms share the same prothrombinase-inhibiting anticoagulant toxicity, this suggests that varespladib binds to a conserved site on the toxin surfaces (and likely the enzymatic pocket itself), whereas the metalloprotease inhibitors exert their effects by binding to highly variable sites that are distinct from the enzymatic pocket and variable across *Bitis* toxins. Furthermore, the binding site to which marimastat and prinomastat bind for the anticoagulant sPLA_2_ toxins present within *Naja* venoms is potentially absent or at least highly divergent from the anticoagulant sPLA_2_ within *Bitis* venoms, despite the toxins being of the same toxin class. This thus provides a testable hypothesis for future work investigating the binding site of these metalloprotease inhibitors to sPLA_2_ toxins, with future work needing to take into consideration the dynamicity of sPLA_2_ toxins.

**Figure 3:**
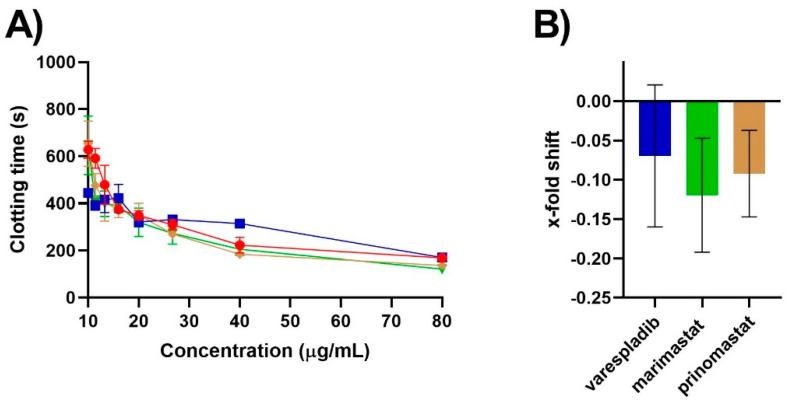
(**A**) Efficacy of varespladib, marimastat and prinomastat at neutralising the fibrinogen-clotting coagulant activity of *B. caudalis* (Rosh Pinah locality) venom. Eight-point concentration curve, representing venom-induced clotting times (red circles), venom pre-incubated with varespladib-Na (blue squares), venom pre-incubated with marimastat (green triangles) and venom pre-incubated with prinomastat (brown diamonds). All values are mean ± SEM of *n* = 3. Note: some error bars are too small to see. (**B**) Percentage drop of plasma clotting time due to pre-incubation with either varespladib (blue), marimastat (green) or prinomastat (brown). All values are mean ± SEM of *n* = 3. For all assays, there was a final reaction concentration of 0.4 nM (varespladib) and 0.2 mM (prinomastat and marimastat).

A notable additional effect was the ability of the Rosh Pinah locale *B. caudalis* venom to also cause clotting in human plasma at concentrations greater than 20 µg/mL (Figure 2A). Previous research has shown this to be a pseudo-procoagulant coagulotoxic activity driven by kallikrein enzymes, which act synergistically with the prothrombinase-inhibiting sPLA_2_ toxins during human envenoming, depleting the available fibrinogen and further potentiating the overall anticoagulant effect of the venom [15]. This direct coagulant activity on fibrinogen has been previously shown to be variable across *B. caudalis*, being most potent in venom from *B. caudalis* of the Namaqualand and Messina localities and absent from the Mariental locality *B. caudalis* [15]. This activity is also well-documented in the venom of other species within both the *Bitis* genus [27,28,29,30], as well as more broadly in the Viperidae family [31,32,33]. Importantly, neither the PLA_2_ inhibitor varespladib or the two metalloprotease inhibitors showed any efficacy at neutralising the kallikrein enzyme-driven venom activity in vitro (Figure 3).

The inability of these inhibitors to neutralise this activity demonstrates that although these inhibitors show a wide range of functionality in neutralising snake venom activities, they are likely unsuitable for completely neutralising venoms rich in kallikrein-type snake venom serine proteinase enzymes. The exact degree to which these sPLA_2_ and kallikrein enzyme toxins act synergistically in vivo remains unstudied and requires investigation. *Bitis caudalis* potentially represents an ideal species to investigate such interactions, since previous research has shown the potency of their sPLA_2_ and kallikrein enzyme-driven anticoagulant actions is also highly variable across different populations [15], which may allow for experiments elucidating toxin interactions to be conducted. Thus, this study highlights an important facet of venom research, which is that multi-drug approaches utilising multiple inhibitors will likely be required, in particular for species containing distinct toxins of various toxin families that act synergistically, to deliver the most effective first-aid treatment conceivable. Although small-molecule enzyme inhibitors that neutralise serine proteinases exist, their suitability as first-aid treatments for snakebites still requires in-depth investigation. One issue is the likelihood of serine proteinase inhibitors to also show promiscuity towards undesired physiological targets, such as human coagulation factors themselves, which are also, primarily, serine proteases. Thus, current research remains primarily directed at investigating the efficacy of PLA_2_ and metalloprotease inhibitors [17,18,21,22,25,34].

Overall, our study further highlights the significant potential but also the limitations SMIs possess as a front-line first-aid treatment for snakebites globally. Delayed treatment following envenoming is known to have severely detrimental effects on patient outcomes. [35,36]. Thus, the potential first-aid use of these SMIs to assist in treatment of snakebites in inaccessible, remote or regional areas where antivenom may be hours or even days of travel away, such as those where the *Bitis* in this study occur, is a major advantage that is currently critically needed to alleviate the global health burden snakebites represent. However, further research into therapeutically viable SMIs that neutralise snake venom serine proteinases such as kallikrein enzymes is required, since neither PLA_2_ nor metalloprotease inhibitors appear effective for this toxin class with respect to the pseudo-procoagulant action of *Bitis* venom. Future work investigating the ability to neutralise the kallikrein-type serine proteases in venoms will have to run controls to ascertain the neutraliation of the normal body blood clotting serine proteases such as thrombin and factor Xa. Indeed this has proven to be a limitation of serine protease inhibitors in neutralising venom serine proteases. Thus, while metalloproteases and PLA_2_ toxins have potential therapeutic options, the therapeutic options for serine protease toxins remain a major unmet need.

## 3. Materials and Methods

### 3.1. Stock Preparation

#### 3.1.1. Venoms

Lyophilised venoms of *B. atropos*, *B. caudalis* (Rosh Pinah locality), *B. cornuta* (Kliensee locality) and *B. cornuta* (Springbok locality) were sourced from the long-term cryogenic collection of the Venom Evolution Laboratory. These venoms were stored until use at −20 °C after being reconstituted to 4 mg/mL concentrated venom stock by adding 1:1 (double deionised) ddH_2_O:glycerol. The concentration was confirmed using a Thermo Fisher Scientific™ NanoDrop 2000 UV–Vis Spectrophotometer (Thermofisher, Sydney, Australia) set to 280 nm wavelength. All venom work was undertaken under the authority of UQ Biosafety Committee approval #IBC134BSBS2015 and University of Queensland Animal Ethics approval 2021/AE000075.

#### 3.1.2. Plasma

Pooled 3.2% citrated recovered plasma was obtained from the Australian Red Cross (Research agreement #18-03QLD-09 and University of Queensland Human Ethics Committee approval #2016000256), aliquoted to 1 mL quantities, flash-frozen in liquid nitrogen and stored at −80 °C. For experimentation, aliquots were defrosted at 37 °C in a Thermo Haake ARCTIC water bath (Thermofisher, Sydney, Australia). Defrosted plasma aliquots were replaced every hour for freshness. All plasma work was undertaken under the UQ Biosafety Committee approval #IBC134BSBS2015.

#### 3.1.3. Enzyme Inhibitors

Three SMIs were included to determine their efficacy against the included *Bitis* venoms. Varespladib (varespladib:Na) was provided by Ophirex, Inc. (Corte Madera, CA, USA). Two metalloprotease inhibitors were purchased from Sigma-Aldrich (St. Louis, MI, USA): (1) prinomastat hydrochloride ((S)-2,2-Dimethyl-4-((p-(4-pyridyloxy)phenyl) sulfonyl) -3-thio- from Sigma-Aldrich (catalogue# PZ0198) and (2) marimastat (2S,3R)-morpholinecarbohydroxamic acid hydrochloride) >95% (HPLC) N4-[(1S)-2,2-Dimethyl-1-[(methylamino)carbonyl]propyl]-N1,2-dihydroxy-3-(2-methylpropyl) butanediamide (catalogue #M2699). All inhibitors arrived in powdered form, were dissolved in 10% dimethyl sulfoxide (DMSO) and further diluted using deionized water to form main stocks of 10 mM concentration. Inhibitor preparation and concentration were chosen based upon previous literature, including that of Chowdhury et al. (2021) [25].

### 3.2. Assay Conditions

Protocols published and validated by Bittenbinder et al. (2018) and Chowdhury et al. (2021) were adapted for this study [20,25]. A STA-R Max^®^ (Stago, Asnières sur Seine, France) coagulation analyser was used to determine venom action in the absence and presence of SMIs on prothrombinase inhibition as well as fibrinogen clotting. The STA-R Max^®^ measures time until clot formation automatically, utilising a viscosity-based detection system that is implemented by the presence of opposing magnets and a metal ball inside the cuvette. A working stock of 800 μg/mL was prepared by diluting 4 mg/mL venom stocks with Owren Koller buffer (Stago catalogue #00360). The working stock was loaded into the analyser for running 8-point concentration curves, with 25 µL of the venom being serially diluted to: 114.3, 57.1, 38, 28.6, 22.9, 19, 16.3 and 14.3 µg/mL concentrations in the 175 µL incubation reaction and 80, 40, 26.6, 20, 16, 13.3, 11.4 and 10 µg/mL concentrations in the 250 µL final reaction volume. For the prothrombinase inhibition assay, the robot added 50 µL 0.025 M calcium (Stago catalogue #00367) + 25 µL of Owren Koller buffer, 50 µL of phospholipid (Stago catalogue #00597) and 75 μL of human plasma. This was followed by a gentle shake-mix and then 2 min of incubation at 37 °C. Then, 25 µL of FXa (Stago catalogue #00311) was added and clotting time was measured immediately. For the fibrinogen-clotting assay, 50 µL of the venom sample was serially diluted to the same concentrations as the previous assay before the robot added 50 µL 0.025 M calcium, 25 µL of Owren Koller buffer and 50 µL of phospholipid. This was followed by a gentle shake-mix and then 2 min of incubation at 37 °C. Then, 75 µL of bovine fibrinogen was added, and clotting time was measured immediately. To avoid venom degradation and maintain true replicates, fresh venom was loaded after each replicate of 8 dilutions. As a negative control test for each assay, a 1:1 (double deionised) ddH_2_O:glycerol blank solution was used in place of venom.

To determine the efficacy of SMIs, the aforementioned 8-point concentration curves were repeated with the addition of SMIs diluted within the 25 µL Owren Koller buffer. For varespladib, the variant was prepared to a concentration 4 nM. For the metalloprotease inhibitors, the concentration used (2 mM) had been shown previously to inhibit metalloproteases and cross-react with PLA_2_ toxins. For each inhibitor, there were an incubation reaction concentrations of 0.0057 nM (varespladib) and 0.29 mM (prinomastat and marimastat) and a final reaction concentrations of 0.4 nM (varespladib) and 0.2 mM (prinomastat and marimastat).

### 3.3. Statistical Analyses

To compare the area under the curve (AUC) of venom and venom + SMI, we calculated an X-fold magnitude of shift (formulae [(AUC of venom incubated with SMI/AUC of venom) − 1]) and, later, generating percentage (multiplying 100 with X-fold shift values) drop of clotting time. Data were analysed using GraphPad Prism 9.1.0 (GraphPad Prism Inc., La Jolla, CA, USA). All raw data are available in the Supplementary Materials. Note that for all assays that reached the maximum machine reading time of 999 s, a value of 999 s was recorded.

## Data Availability

All raw data are available within the Supplementary Materials.

## Supplementary Materials

All raw data can be downloaded from the supplementary file.
